# Supplementary material for: Pre-Hospital Emergency Medical Services Utilization Amid COVID-19 in 2020: Descriptive Study Based on Routinely Collected Dispatch Data in Bavaria, Germany
Source: Healthcare (Basel). 2023 Jul 8;11(14):1983. doi: 10.3390/healthcare11141983 (PMC10379196; doi:10.3390/healthcare11141983)

1 Supplement Table S1: Timeline of key COVID-19 mitigation measures in Bavaria …………...2

2 Supplement Table S2: Description of dispatch keywords……………………………………...4

3 Supplement Figure S1: Emergency rates 2020 and average rates 2018/2019…………….……5

4 Supplement Figure S2: Transport rates 2020 and average rates 2018/2019…………………...…6

Supplement Table S1. Timeline of key COVID-19 mitigation measures in Bavaria

Dates	Key aspects	Schools	Daycare / kindergarten
Frist lockdown 20.03.2020 – 10.05.2020<br><br>(state of emergency 20.03.2020 – 15.06.2020)	stay-at-home order: leaving one's home is permitted for essential purposes only<br><br>citizens are requested to reduce contact with others, except members of their own household, to an absolute minimum.<br><br>no more than one household or two people not living in the same household are allowed to meet in private or public<br><br>non-essential businesses and restaurants are closed, non-essential activities are prohibited, delivery and pick up of takeaway food is permitted	schools are closed from 16.03 – 10.05.2020<br><br>distance learning is established<br><br>closures partially exclude graduating classes and emergency childcare	daycare centers and kindergartens are closed from 16.03 – 10.05,<br><br>emergency childcare is offered for children of parents working in certain occupational groups (i.e. critical infrastructure)
Incidence-related restrictions 11.5.2020 - 01.11.2020<br><br>"traffic light system" from 17.10.2020	no movement restrictions<br><br>stores are reopen, cultural events and leisure activities are allowed<br><br>citizens are still requested to reduce contact with those outside of their own household to a minimum, yet contact restrictions are loosened and private events can be held<br><br>mask mandates in certain locations<br><br>incidence-related restrictions with the introduction of a "traffic light system"	face-to-face teaching with suitable measures to ensure that a minimum distance of 1.5 m can be maintained during class	daycare centers and kindergartens are open with hygiene concepts in place
Lockdown "light" 02.11.2020 – 09.12.2020	up to 10 people from no more than two households can meet<br><br>non-essential shops stay open<br><br>culture and leisure services as well as restaurants are closed, delivery and pick up of takeaway food is permitted	for each school, local authorities decide whether face-to-face teaching is feasible	daycare centers and kindergartens stay open to everyone in need of childcare

Second lockdown 09.12.2020 – 18.04.2021<br><br>(state of emergency 09.12.2020 – 06.06.2021)	stay-at-home order: leaving one's home is permitted for essential purposes only<br><br>curfews in 'hotspots' (7 day incidence of >200 cases/100,000 population) from 21:00-05:00<br><br>up to 10 people from no more than two households can meet<br><br>non-essential businesses and restaurants are closed, non-essential activities are prohibited, delivery and pick up of takeaway food is permitted, goods can be pre-ordered and picked up from retail stores	schools are closed and distance learning is in place from 16.12, some children are eligible for emergency childcare in schools	daycare centers and kindergartens are closed from 16.12, emergency childcare is offered

Supplement Table S2. Description of dispatch keywords

Category	Description
Pain	Pain (acute or increasing), severe pain, sudden onset of severe headache or colicky pain
Respiratory	Shortness of breath, dyspnea, (acute), increasing respiratory dysfunction (cyanosis, stridor, wheezing, bradypnea, tachypnea, respiratory arrest)
Consciousness	Disturbance of consciousness, unconsciousness
Cardiovascular	Cardiovascular complaints (acute), hyper- / hypotension, acute onset of chest pain, shock signs, bradycardia, arrhythmia, pacemaker malfunction, allergic reaction
Pediatric	Child sick or injured (classification as pediatric is not depending on age, but on the dispatcher's classification)
Neurologic	Unspecified symptoms and signs involving cognitive function and awareness, neurological deficits including disturbance of consciousness, seizure
Mental health	Psychiatric condition that does not allow referral to primary care, suicide risk, situation with third party at risk
Other emergency	Metabolic disturbances, intoxication, gastrointestinal/gynecologic bleeding, nosebleed, abnormal body temperature, home emergency call, pregnancy-related conditions
Trauma	Acute injury (with and without instable vital signs)
Traffic accident	Acute injuries with the need for prompt care after traffic accidents (with and without instable vital signs)

Supplement Figure S1. Emergency rates 2020 and average rates 2018/2019

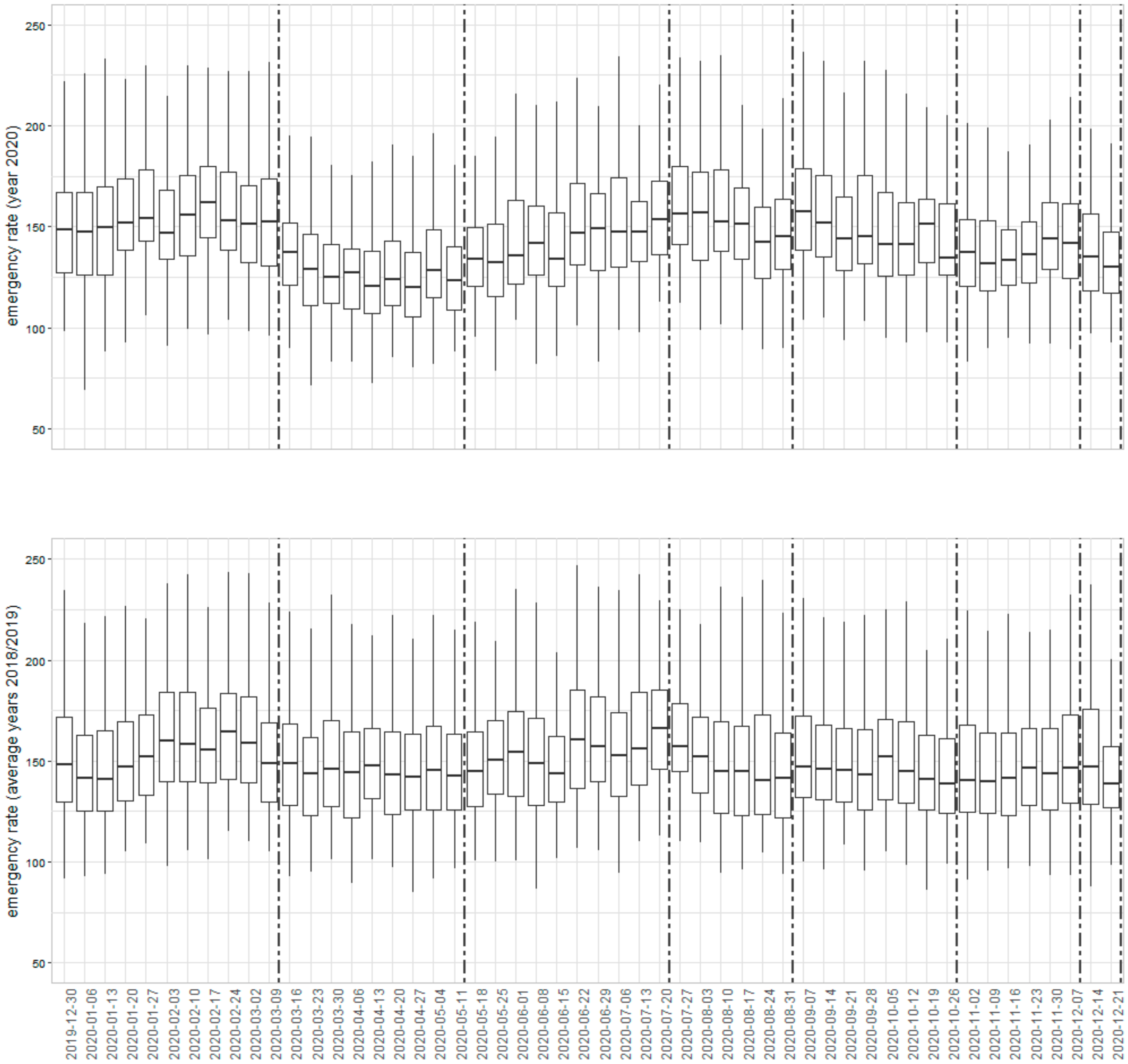

Supplement Figure S2. Transport rates 2020 and average rates 2018/2019

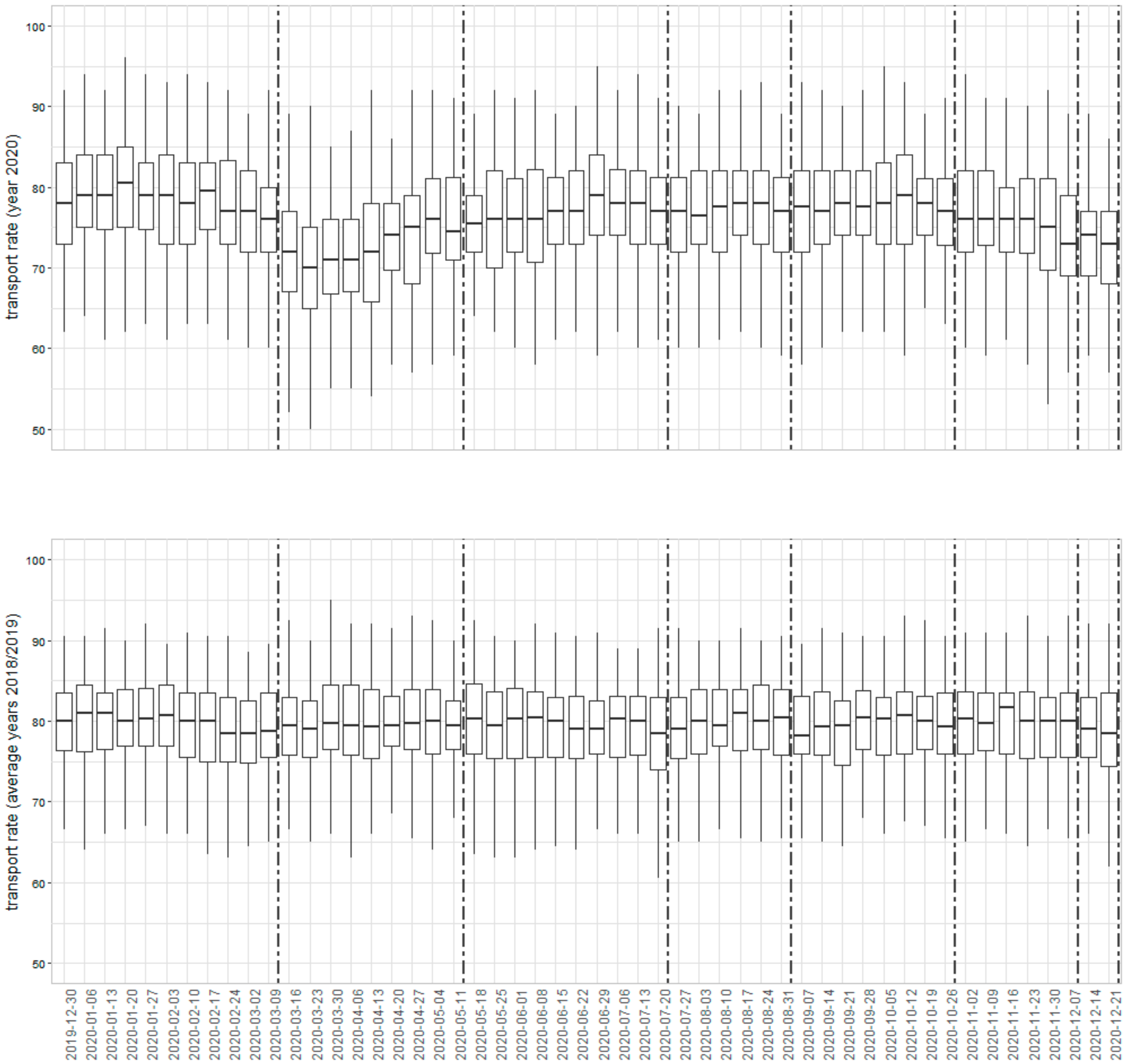

Supplement: Supplementary file 1 [file healthcare-11-01983-s001.zip › healthcare-2444190-supplementary.pdf]
